# Simultaneous Oblique Lumbar Interbody Fusion and Percutaneous Pedicle Screw Fixation Combined With Endoscopic-Assisted Decompression in the Lateral Position for Two-Level Lumbar Pathology

**DOI:** 10.7759/cureus.108565

**Published:** 2026-05-09

**Authors:** Arvind Umarani, Masato Tanaka, Tadashi Komatsubara, Ryou Ugawa, Bishnu D Sharma

**Affiliations:** 1 Orthopaedic Surgery, Okayama Rōsai Hospital, Okayama, JPN

**Keywords:** endoscopic decompression, lateral position, o-arm navigation, oblique lumbar interbody fusion, percutaneous pedicle screw fixation

## Abstract

Hybrid minimally invasive spine surgery (MISS) is evolving with the integration of navigation and endoscopic techniques. We describe a fluoroscopy-free hybrid MIS procedure for pathology at two different spinal levels.

A 71-year-old female with recurrent low back and left leg pain following prior multilevel decompression underwent oblique lumbar interbody fusion (OLIF) with simultaneous percutaneous pedicle screw (PPS) fixation and lateral endoscopic-assisted decompression at separate levels without repositioning and without intraoperative fluoroscopy.

The operative time was 163 minutes, with 200 mL of estimated blood loss (EBL) and no intraoperative complications. Postoperative imaging confirmed accurate screw placement with adequate neural decompression, both direct and indirect. The Oswestry Disability Index (ODI) improved from 49% to 20% at the one-year follow-up.

This technical note demonstrates the feasibility of a simultaneous two-surgeon workflow for OLIF, with drift-prevention steps and endoscopic-assisted decompression in the same position, to reduce radiation exposure, blood loss, and operative time while providing efficient and safe treatment for selected lumbar degenerative cases. This case is particularly important in situations in which the patient requires decompression in the lateral decubitus position, such as during pregnancy or in cases of severe chest or abdominal injury.

## Introduction

As minimally invasive spine surgery (MISS) continues to expand, modern navigation and endoscopic methods are enabling a shift toward a fluoroscopy-free workflow that enhances surgeon safety [1]. Traditional oblique lumbar interbody fusion (OLIF) procedures rely on intraoperative fluoroscopy for level localization and pedicle screw placement, often necessitating patient repositioning from the lateral to the prone position [2]. These additional steps increase surgical time, radiation exposure, and contamination risk.

Spine surgeons experience among the highest levels of occupational fluoroscopy exposure due to the frequent use of fluoroscopic imaging, increasing their risk of cancer, cataracts, and other radiation-related health problems [3]. These concerns have driven the adoption of navigation-based workflows that reduce or eliminate intraoperative fluoroscopy.

With the introduction of O-arm-based navigation systems, spinal fusion and percutaneous pedicle screw (PPS) fixation can be performed accurately in a single lateral position [4]. Parallel advancements in endoscopic spine surgery have made direct decompression more feasible [5]. In two-level lumbar stenosis, where one level requires fusion and another requires decompression, a hybrid minimally invasive surgery (MIS) approach offers an efficient solution [6]. In this article, we report a C-arm-free, lateral-position OLIF procedure integrated with simultaneous PPS fixation and endoscopic decompression, which may offer workflow and ergonomic advantages over conventional MIS-transforaminal lumbar interbody fusion (TLIF) in selected cases.

## Case presentation

The Ethics Committee of Okayama Rōsai Hospital approved this case report (Approval No. 575). Written informed consent was obtained from the patient. This technique is indicated for patients who require lumbar interbody fusion and direct decompression without a change in position. The best indications for this technique are patients in the late stages of pregnancy or those with severe chest or abdominal injury.

A 71-year-old female with a past history of rheumatoid arthritis complained of low back and left lower limb radicular pain that had begun 4 years earlier and had progressively worsened. Her walking distance was significantly reduced, with a claudication distance of <200 meters. She had previously undergone L2-3 to L5-S1 posterior decompression surgery at another hospital 3 years earlier and had been receiving conservative treatment for the same condition.

**Figure 1:**
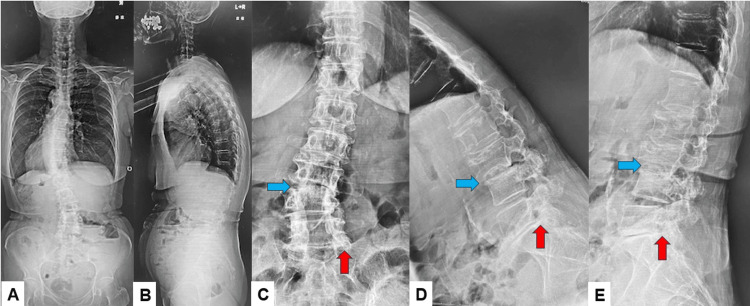
Preoperative radiographs. Preoperative radiographs showing L3-4 collapse with associated endplate changes. A: Anteroposterior whole-spine radiograph; B: lateral whole-spine radiograph; C: anteroposterior lumbar radiograph; D: lateral flexion lumbar radiograph; E: lateral extension lumbar radiograph. Red arrows indicate left foraminal narrowing. Blue arrows indicate L3/4 angulation and instability.

Physical examination revealed a healed midline scar with tenderness in flexion and extension positions. There was mild muscle weakness of the left extensor hallucis longus muscle (MMT 4/5) and hypoesthesia in both L5 dermatomes. Standing whole spine and lumbar radiographs demonstrated L3-4 collapse with associated endplate changes (Figure 1).

**Figure 2:**
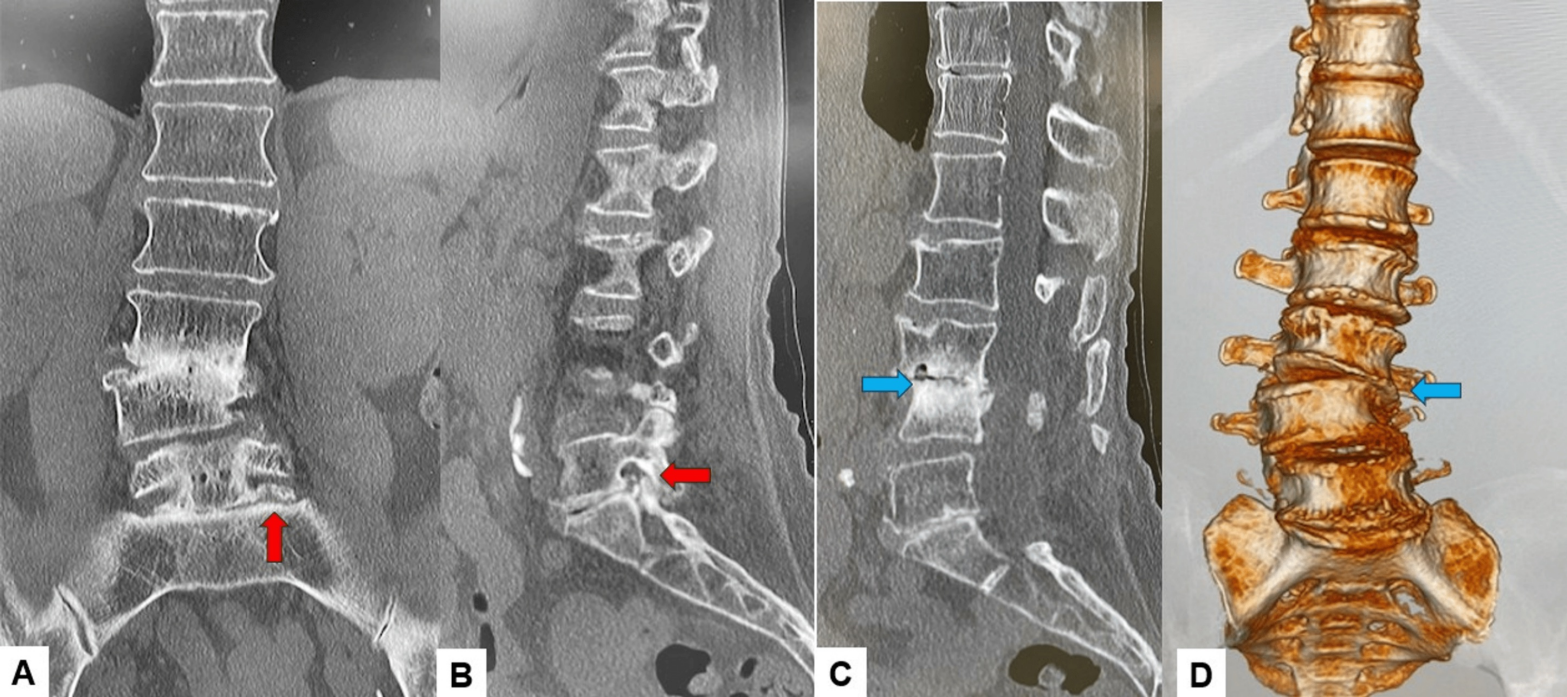
Preoperative CT. A: Coronal reconstructed CT image; B: left parasagittal reconstructed CT image; C: midsagittal reconstructed CT image; D: 3D CT image. Red arrows indicate left foraminal stenosis. Blue arrows indicate L3/4 angulation and osteoarthritic changes.

**Figure 3:**
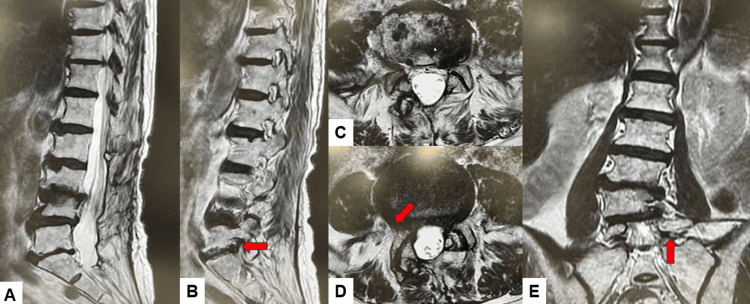
Preoperative MRI. A: Midsagittal T2-weighted image; B: left parasagittal T2-weighted image; C: axial T2-weighted image at the L4/5 level; D: axial T2-weighted image at the L5/S1 level; E: coronal T2-weighted image. Red arrows indicate left L5/S1 foraminal stenosis.

CT of the lumbar spine revealed left L5/S1 foraminal stenosis, L3/4 angulation, and degenerative osteoarthritic changes (Figure 2). MRI showed severe compression of the left L5 nerve root at the left L5/S1 neural foramen (Figure 3).

**Figure 4:**
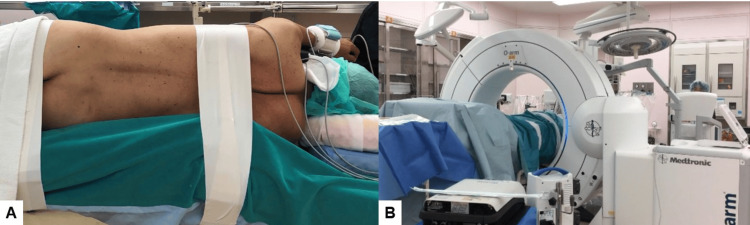
Patient positioning and O-arm imaging. A: Patient positioning in the lateral decubitus position. B: O-arm docking and 3D image acquisition for navigation. The operating table is flexed approximately 20° in convexity to open the disc space.

The patient was positioned in the right lateral decubitus position on a radiolucent Jackson frame with the ProAxis spine table (Mizuho OSI, USA), with the table flexed approximately 20° in convexity (Figure 4A). Neuromonitoring leads were connected, and baseline signals were recorded.

A percutaneous reference frame was fixed 2 cm anterior to the left posterior superior iliac spine (PSIS), and an intraoperative O-arm scan (Medtronic, USA) was performed for navigation registration on the StealthStation S8 platform (Medtronic, USA) (Figure 4B). Accuracy was rechecked at critical stages, and the reference frame was shielded from accidental contact during subsequent procedures.

**Figure 5:**
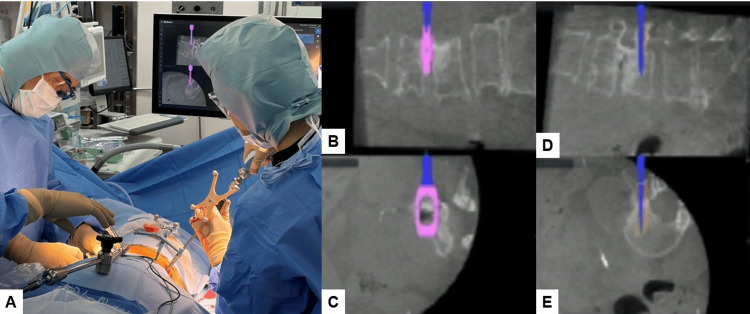
C-arm-free simultaneous OLIF and PPS. A: Intraoperative image; B: coronal navigation image with cage; C: axial navigation image with cage; D: sagittal navigation image with pedicle probe; E: axial navigation image with pedicle probe. OLIF: Oblique lumbar interbody fusion; PPS: Percutaneous pedicle screw.

A two-surgeon workflow was used (Figure 5A), with the first surgeon performing OLIF and the second simultaneously placing PPS. Using a navigated pointer, a skin incision was made approximately 3-4 cm lateral to the anterior disc margin. With blunt dissection, the retroperitoneal space was gently entered, and the psoas muscle was retracted posteriorly under direct visualization. Navigated dilators and retractors were then docked onto the disc space. Disc preparation and cage insertion were performed. A titanium-coated polyetheretherketone (PEEK) cage packed with bone graft was inserted obliquely across the disc space (Figures 5B-5C).

**Figure 6:**
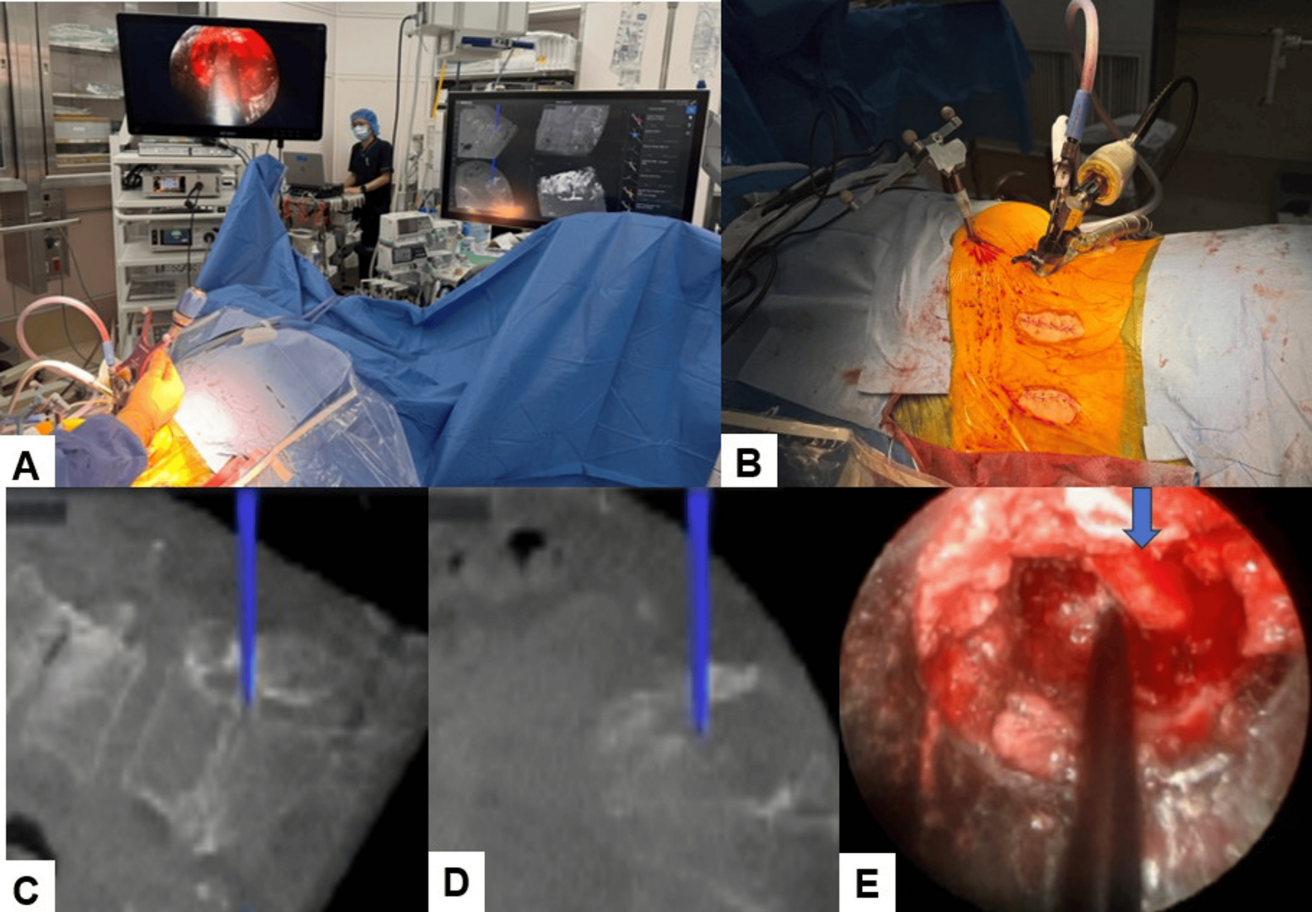
Intraoperative images. A: Surgeon standing on the patient’s dorsal side for the lateral microendoscopic approach; B: METRx tubular retractor with an 18-mm working channel docked at L5-S1 under navigation guidance; C: navigation-guided pointer localizing the target in the lateral view; D: navigation-guided pointer localizing the target in the axial view; E: navigation confirmation of the bony resection limits and adequate decompression.

After the navigated OLIF, the surgeon stood on the patient’s dorsal side (Figure 6A). The METRx system (Medtronic, Minneapolis, MN, USA) was used to perform decompression through an 18-mm working channel placed under navigation at the stenotic adjacent level (Figure 6B). Decompression was achieved using an endoscopic high-speed burr (Midas Rex, Medtronic, USA), ensuring complete decompression of the left L5 nerve root (Figures 6C-6E).

No intraoperative complications were observed during the procedure. The operative time was 163 minutes, and the estimated blood loss (EBL) was 200 mL.

**Figure 7:**
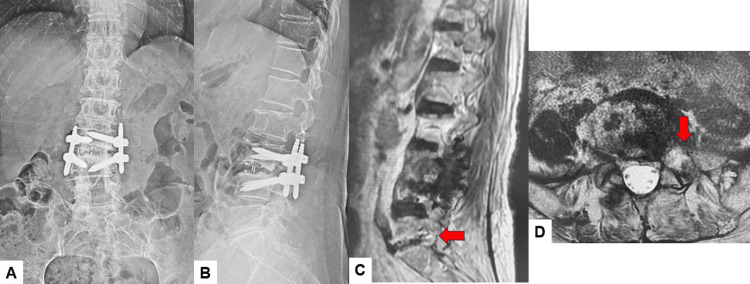
Postoperative radiographs and MRI. A: Anteroposterior lumbar radiograph; B: lateral lumbar radiograph; C: left parasagittal T2-weighted image; D: axial T2-weighted image at the L5/S1 level. Red arrows indicate adequate nerve decompression.

Postoperative radiographs and MRI confirmed accurate pedicle screw placement in all pedicles, with adequate neural decompression (Figure 7). The patient reported marked improvement in left L5 radicular pain, with resolution of preoperative numbness on standing. The patient was mobilized with a brace on postoperative day 1. No new neurological deficit or perioperative complication was observed.

**Figure 8:**
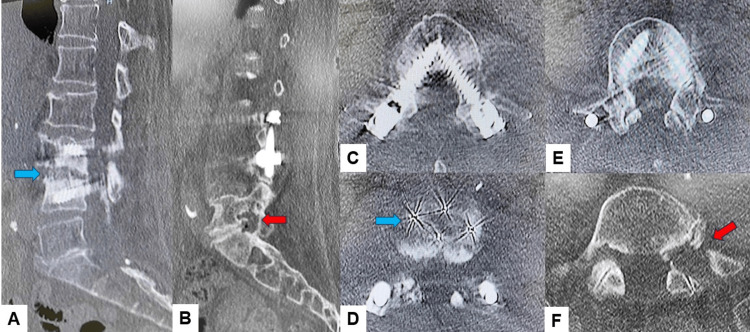
Final follow-up CT. A: Midsagittal reconstructed CT image; B: left parasagittal reconstructed CT image; C: axial CT image at L3; D: axial CT image at L3/4; E: axial CT image at L4; F: axial CT image at L5/S1. Blue arrows indicate the OLIF cage. The red arrow demonstrates adequate decompression of the left L5 exiting nerve root. Postoperatively, lumbar lordosis improved from 25° to 32°, and foraminal height increased from 6.8 mm to 9.4 mm, respectively. OLIF: Oblique lumbar interbody fusion.

At the three-month follow-up, she was able to walk more than 1 km without claudication. Sensory disturbance in the L5 distribution had almost completely resolved, with intact neurology. At the one-year follow-up, her Oswestry Disability Index (ODI) score decreased from 49% to 20%. CT confirmed appropriate cage position, restoration of disc and foraminal height, and evidence of progressive fusion at the L3-4 level, with no cage subsidence or adjacent segment degeneration. Postoperatively, lumbar lordosis improved from 25° to 32°, and foraminal height increased from 6.8 mm to 9.4 mm, respectively (Figure 8).

## Discussion

The field of MISS continues to advance with the widespread adoption of navigation and endoscopic technologies [7]. In this setting, careful selection of the most appropriate approach for each specific pathology is essential to optimize outcomes. A true MIS hybrid technique integrates two complementary minimally invasive procedures within a single workflow [6]. Our technique aims to reduce complications while enhancing both surgeon safety and patient recovery.

In our patient, the radicular pain was primarily attributable to foraminal stenosis at L5-S1, whereas bony distraction and angulation at L3-4 were identified as the main generators of low back pain. Based on this, L3-4 fusion with targeted decompression of the left L5-S1 foramen was considered the most appropriate treatment strategy. Performing navigation-guided OLIF in the supine position, along with endoscopy, would require an additional position change. Another option would have been single-prone-position MIS-TLIF with decompression at L5-S1. This could have avoided separate decompression at L5-S1, but it requires paraspinal muscle dissection and allows insertion of a smaller interbody cage than that used in OLIF [8]. The hybrid approach was chosen to avoid posterior redissection in a previously decompressed spine and to address two distinct problems in a single position while facilitating placement of a large-footprint cage, which can be advantageous for achieving superior fusion rates, restoring lumbar lordosis, and reducing the risk of cage subsidence, while also minimizing muscle trauma.

An additional strength of our technique lies in its workflow efficiency and radiation profile. In terms of workflow efficiency, performing the procedure in a single lateral position using navigation avoids the need for intraoperative repositioning, which can otherwise add operative time compared with conventional dual-position OLIF [2]. In terms of radiation profile, our C-arm-free setup eliminates intraoperative fluoroscopy and may reduce occupational fluoroscopy exposure to the surgical team, addressing a growing concern regarding cumulative occupational radiation in spine surgery. Performing PPS in the same lateral position as OLIF has also been shown to improve surgical efficiency and reduce the risk of infection and contamination by eliminating redraping and limiting repositioning [2].

Endoscopic fusion has gained popularity in recent years. It could have been an option in our case, as it allows decompression and fusion through small incisions with excellent visualization and minimal muscle trauma [9]. However, it has its own limitations, including limited bone graft volume, the need for a small-footprint cage, longer operative times, a steep learning curve, and relatively higher reported nonunion rates compared with lateral or open fusion techniques [9].

Prone transpsoas (PTP) is another minimally invasive option that enables lateral interbody work and posterior fixation in a single prone position, avoiding intraoperative repositioning and preserving musculature [10]. Nevertheless, open conversion in PTP can be technically challenging, and the transpsoas corridor carries a risk of lumbar plexus irritation and potential vascular or visceral injury [11]. A comparison of all the aforementioned techniques with our technique is summarized in Table 1.

**Table 1:** Conceptual comparison of workflow characteristics across different approaches: non-comparative and literature-informed. Note: Reference numbers in brackets indicate the sources supporting specific statements within the table. OLIF: Oblique lumbar interbody fusion; PPS: Percutaneous pedicle screw; MIS-TLIF: Minimally invasive transforaminal lumbar interbody fusion; MIS: Minimally invasive surgery; TLIF: Transforaminal lumbar interbody fusion.

Approach	Advantage	Disadvantage
Our technique: C-arm-free OLIF + simultaneous PPS with lateral endoscopy (Lateral position)	Indirect + direct decompression No radiation exposure Better fusion [12] Good lordosis and good union rate Possible at L5-S1	Vascular/visceral injury [12] Unfamiliar position for endoscopy
MIS-TLIF with decompression (Prone position)	Direct decompression [12] No major vascular/visceral risks	Due to the small-footprint cage, relatively high nonunion rates [12] Dural injury Paraspinal muscle splitting
Endoscopic fusion with endoscopic decompression (Prone position)	Direct decompression Good visualization Smaller surgical scar Preservation of anatomic planes	Long operative time [9] Learning curve [9] Due to the small-footprint cage, relatively high nonunion rates and difficulty in achieving good lordosis
Prone transpsoas approach with endoscopic decompression (Prone position)	Indirect decompression Good lordosis Shorter operative time	Difficult open conversion [10] Lumbar plexus injury [10] Vascular/visceral injury [10] Not possible at L5-S1

In our C-arm-free technique, EBL and surgical time were 200 mL and 163 minutes, respectively. Oda Y et al. demonstrated that integrating lateral endoscopic decompression into a single-position workflow permits fusion at one level and decompression at another without the need for repositioning or fluoroscopy [13]. Kapoor S et al. demonstrated that microendoscopic discectomy can be safely performed in the lateral position in pregnant patients for whom prone positioning is contraindicated [14]. More broadly, the lateral position helps mitigate risks associated with prone positioning, such as postoperative visual loss and lateral femoral cutaneous neuropathy. It also simplifies cardiopulmonary resuscitation should cardiac arrest occur in the operating room [15]. Furthermore, the lateral position offers physiological advantages, including reduced abdominal pressure and decreased intraoperative blood loss [16].

Compared with other techniques, our procedure yielded better or comparable results. Single-position OLIF and PPS without second-level decompression has been reported with 270 ± 238 mL of EBL, a surgical time of 198 ± 41 minutes, and 3.3 ± 1.2 minutes of C-arm time [11]. MIS-TLIF has been reported with 181 ± 225 mL of EBL, 259 ± 43 minutes of surgical time, and 2.4 minutes of C-arm usage [17]. Transforaminal endoscopic fusion without second-level decompression had less EBL and a similar surgical time, but C-arm use was necessary [18].

Navigation-guided endoscopic decompression provides 3D accuracy for working-channel or tube placement, bone resection, and root decompression while eliminating fluoroscopy exposure [19]. Mehta R et al. described navigation-guided endoscopic decompression for the L5-S1 foramen, showing that O-arm navigation enables accurate tube placement, precise bone removal, and safe root decompression while eliminating C-arm radiation [20]. The use of a navigated burr ensures controlled facet resection and minimizes the risk of postoperative instability [7]. This helped us maintain segmental stability at L5-S1, thereby reducing the risk of postoperative instability.

There are several limitations to our technique. First, the absolute indication for this technique is limited. Second, endoscopic decompression in the lateral position is somewhat technically demanding. Third, expensive equipment, such as the O-arm and spinal navigation system, is recommended for this technique.

In the present case, since both OLIF and PPS fixation were performed simultaneously in the lateral position, extending the workflow to incorporate endoscopic decompression in the same orientation provided a seamless, minimally invasive, and time-efficient solution. This strategy eliminated the need for repositioning and further minimized anesthesia duration. These findings support the rationale for a hybrid, single-position, navigation-assisted approach in lumbar degenerative pathology.

## Conclusions

This case report demonstrates the feasibility of a single-position, navigation-guided hybrid workflow without intraoperative fluoroscopy, which may represent a safe, efficient, ergonomic, single-position solution for managing complex lumbar canal stenosis with instability. The integration of O-arm-based navigation enhances intraoperative accuracy, may reduce operative complications, and minimizes radiation exposure for both the patient and the surgical team. This hybrid MIS approach exemplifies the progressive direction of modern MISS by combining precision, safety, and workflow efficiency within a single, integrated platform. However, this is a single-case experience, and further validation is needed.
